# Immersive Versus Non-immersive Experience: Exploring the Feasibility of Memory Assessment Through 360° Technology

**DOI:** 10.3389/fpsyg.2019.02509

**Published:** 2019-11-14

**Authors:** Sara Ventura, Eleonora Brivio, Giuseppe Riva, Rosa M. Baños

**Affiliations:** ^1^Department of Personality, Assessment and Psychological Treatments, University of Valencia, Valencia, Spain; ^2^Department of Psychology, Centro Studi e Ricerche di Psicologia della Comunicazione, Università Cattolica del Sacro Cuore, Milan, Italy; ^3^Applied Technology for Neuro-Psychology Laboratory, Auxologico Institute, Milan, Italy; ^4^CIBERObn Ciber Physiopathology of Obesity and Nutrition, Madrid, Spain

**Keywords:** panorama technology, 360° camera, memory assessment, episodic memory, case-comparison

## Abstract

Episodic memory is essential to effectively perform a number of daily activities, as it enables individuals to consciously recall experiences within their spatial and temporal environments. Virtual Reality (VR) serves as an efficacious instrument to assess cognitive functions like attention and memory. Previous studies have adopted computer-simulated VR to assess memory, which realized greater benefits compared to traditional procedures (paper and pencil). One of the most recent trends of immersive VR experiences is the 360° technology. In order to evaluate its capabilities, this study aims to compare memory performance through two tasks: immersive task and non-immersive task. These tasks differ based on the participant’s view of the 360° picture: (1) head-mounted display (HMD) for immersive task and (2) tablet for non-immersive task. This study seeks to compare how memory is facilitated in both the 360° immersive picture as well as the non-immersive 360° picture. A repeated measure design was carried out in a sample of 42 participants, randomized into two groups of 21. Group 1 first observed Picture A (immersive) followed by Picture B (non-immersive) while Group 2 began with Picture B and then looked at Picture A. Each 360° picture contains specific items with some items appearing in both. Memory evaluation is assessed immediately after the exposure (recall task), then again after a 10-min delay (recognition task). Results reveal that Group 1, which began with the immersive task, demonstrated stronger memory performance in the long term as compared to Group 2, which began with the non-immersive task. Preliminary data ultimately supports the efficacy of the 360° technology in evaluating cognitive function.

## Introduction

In the last few decades, Virtual Reality (VR) has shown to be an efficacious instrument in assessing cognitive functions such as attention, memory, and executive functions. VR is defined as an advanced form of human-computer interfaces that allow the user to interact with and be immersed in a virtual environment that reflects reality (Schultheis and Rizzo, 2001).

Researchers have demonstrated that VR-based intervention methods have several important benefits as compared to traditional methods. Traditional methods consist of a set of predefined stimuli delivered in a controlled environment *via* paper-and-pencil or computer systems (Wilson et al., 1989; Negut et al., 2016); however, they have only a moderate level of ecological validity in predicting real-world performance (Alvarez and Emory, 2006; Parsons, 2015). To overcome this limitation, researchers have adopted VR approaches to develop neuropsychological programs that evaluate participants in situations as close as possible to real life (Parsons and Rizzo, 2008; Parsons, 2015; Gamito et al., 2017; Parsons and Barnett, 2017). Specifically, a number of VR platforms have been developed to study and train memory abilities (Castelnuovo et al., 2003; Armstrong et al., 2013; Gamito et al., 2016; Lee et al., 2018). These virtual environments allow users to simulate daily activities such as purchasing products from a shopping list or recalling a route from one point to another within a virtual city (Dores et al., 2012; Parsons and Barnett, 2017). Although results have been encouraging, highlighting the advantages of using VR for cognitive assessment (Faria et al., 2016; Gamito et al., 2018), the development of these virtual environments requires a high economic investment, both in software and hardware requirements.

One recent trend in technology field is the 360° technology (Huang et al., 2017). The 360° camera can record the environment in all directions, allowing users to look up and down, left and right, as he or she can do in real-life situations. More so, it is affordable and does not require any specific technical skills for basic use (Parsons, 2015; Serino et al., 2017). Because of its versatility, the 360° technology has been used in various fields like education (McKenzie et al., 2019), immersive journalism (Schutte and Stilinović, 2017), and advertising (Habig, 2016). This paper presents a case study of the feasibility of alternative approaches to create VR content for memory assessment. Specifically, instead of creating graphics-based VR scenarios (Slater and Sanchez-Vives, 2016), the 360° technology was used to record a familiar environment before playing it to participants on a head-mounted display (HMD).

The 360° videos can be experienced through immersive and non-immersive media (Negro-Cousa et al., 2019). Immersive and non-immersive media vary based upon the participant’s point-of-view and the experience produced during use. Through immersive 360° technology, participants can view the full panorama with the HMD VR support, creating a high sense of presence and immersion, as if they are essentially inside the environment. Through non-immersive 360° environments, participants can view the 360° panorama content by moving or rotating the device in which the content is played, such as a PC, smartphone, or tablet (Repetto et al., 2018). In this view, participants are only external observers. The difference between immersive and non-immersive environments can be better clarified through the concept of spatial presence. Spatial presence is defined as “the sense of being in an environment” (Kober et al., 2012). Immersive 360° environments allow participants to feel as though they are inside the environment while non-immersive environments only allow participants to see the contents based on how the device in use – PC, smartphone, or tablet – is held and moved.

Robertson et al. (2016) adopted an immersive condition for memory evaluation and investigated how visual images are represented by the brain. The authors found that as participant views a panorama picture, the images processed in his or her brain are merged to create the perceptual experience of a coherent continuous panorama. This likely occurs because immersive 360° picture or video elicits the sense of realism and the feeling of being submerged into the environment (Serino and Repetto, 2018). Subsequently, this study aims to investigate the efficacy of 360° panorama technology in strengthening memory function. Specifically, we hypothesized that participants in the 360° immersive condition could better perform in memory tasks than those in the 360° non-immersive condition.

The study was approved by The Ethics Committee of Valencia University (Spain), with registration number: H1543407702114.

## Materials and Methods

### Participants

A total of 42 participants were recruited from students and faculty at the University of Valencia. The sample size has been determined by G*Power 3 software (Faul et al., 2007) and a total of 21 participants per group has been estimated, taking into account an alpha error of 0.05, a statistical power of 0.80, and the effect size was found in a previous study (Negro-Cousa et al., 2019).

The inclusion criteria were as follows: (1) ages 18 years and older; (2) proficiency in Spanish; and (3) without physical limitations, especially in the neck or back, that could prevent free body movements. All participants were volunteers and signed the informed consent document before participation in the study, in accordance with the Declaration of Helsinki.

### 360° Pictures and Apparatus

**Figure 1:**
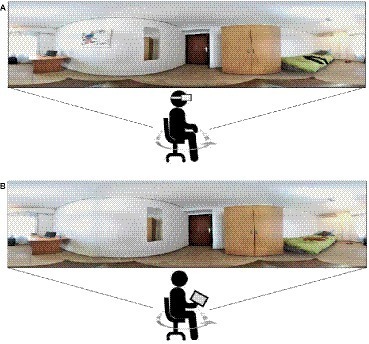
Study task. **(A)** Participants look at the 360° panorama picture through VR headset (immersive condition); **(B)** participants look at 360° panorama picture through tablet (non-immersive condition).

The 360° VR environments were composed of two 360° pictures of bedrooms containing different sets of common household items. Bedroom A (Figure 1A) featured the following target objects: backpack, painting, trousers, shoes, and bottle; Bedroom B (Figure 1B) has the following target objects: glass, T-shirt, slippers, luggage, and portrait. The concepts depicted in each picture according to the objects placed within it are familiar. Familiarity has been shown to have an important effect on various memory- and cognitive-processing tasks. According to Alario and Ferrand (1999), familiarity was rated as an important predictor of picture-naming latencies. Essentially, the naming time is faster when a participant is more familiar with a concept. For example, the bottle in *Bedroom A* was replaced with a vase in *Bedroom B,* or a backpack was replaced with a luggage. Additionally, both pictures have five complementary objects: bed, table, personal computer, mirror, and wardrobe (Sauzéon et al., 2012; Sauzeon et al., 2016; Negro-Cousa et al., 2019). To generate the 360° pictures, the LG360-105 camera and the linked LG360 viewer software for the edit were used. The non-immersive 360° picture was displayed on a Samsung Galaxy Tab A 25.65 cm (10.1″) Tablet, and the immersive 360° picture was displayed on an iPhone 6 *via* a VR head-mounted display (HMD).

### Procedure

Consenting participants in the study were randomly assigned to one of two conditions using the Random Allocation Software 2.0 (Saghaei, 2004). A within-subject counterbalanced design was used: Group 1 first viewed Picture A (immersive) followed by Picture B (non-immersive), and Group 2 first viewed Picture B (non-immersive), then Picture A (immersive) (Figure 1). The counterbalanced design was adopted to avoid the complications connected to a repeated presentation of similar stimuli, and to avoid the primary effect of memory. In this study, the sample was recruited from a non-clinical population with no declared memory deficit, and it was hypothesized that the counterbalanced design could emphasize the difference between the immersive and non-immersive conditions. For participants without memory impairments, it could be easy to remember a list of objects regardless the type of the condition; however, the counterbalanced design was used to minimize the carryover effect (Sayette et al., 2010) as participants perform in more than one condition (immersive and non-immersive or non-immersive and immersive) where the first picture allows for better recollection of the second.

The study took place at the Department of Psychology at the University of Valencia. Participants came to the laboratory, signed the written informed consent, and filled out the sociodemographic questionnaire. Participants were then invited to sit on a swivel chair to ensure the greatest comfort while viewing the 360° picture – through either the HMD for the immersive picture or on the tablet for the non-immersive picture. For the immersive condition, participants looked at a neutral picture in order to focus the image as well as to adjust and calibrate the HMD before displaying the experimental picture. The experiment is composed of three phases: (1) encoding phase, (2) recall phase, and (3) recognition phase (Table 1). During the encoding phase, participants first viewed Picture A or B, depending on the condition they were assigned, for 50 s, then completed the free recall task. Then they viewed the second picture followed by completion of the free recall task again. A 10-min delay was implemented before participants completed the recognition task, where they utilized a written list to identify as many items as they could from the first picture viewed. This procedure (Sauzéon et al., 2012) allowed for best observing the effect of immersive and non-immersive setups for memory ability.

**Table 1:** Counterbalanced design.

Group 1	Group 2
1. View bedroom A (Figure 1A)	1. View bedroom B (Figure 1B)
2. Free recall task	2. Free recall task
3. View bedroom B (Figure 1B)	3. View bedroom A (Figure 1A)
4. Free recall task	4. Free recall task
5. Questionnaires	5. Questionnaires
6. Recognition task of bedroom A (Figure 1A)	6. Recognition task of bedroom B (Figure 1B)

### Measures

#### Sociodemographic Questionnaire

An *ad hoc* questionnaire was created to collect information about each participant’s age, gender, highest education level attained, and physical abilities. This questionnaire was administered as part of the screening process for the study.

#### Memory Failure of Everyday Questionnaire

This is a 28-item questionnaire that measures memory forgetfulness in daily life, rated on a 0–2 scale (0 = never, 1 = sometimes, 2 = many times) (Carrasco et al., 2012). The scale includes three subscales: (1) activities memory, (2) recognition, and (3) communication monitoring. This questionnaire was administered to document possible memory deficits. Higher scores indicate low memory ability. The Spanish adaptation of the scale was used with an adequate internal consistency for the total score (*α* = 0.87) (Montejo et al., 2014).

#### Simulator Sickness Questionnaire

This a 16-item questionnaire that measures the level of discomfort perceived during the 360° immersive condition (Kennedy et al., 1993). The scale includes two subscales: (1) nausea and (2) ocular-motor sickness. It is an adaptation to Spanish from the original scale, reviewed by a bilingual English-Spanish researcher.

#### Computer Experience

The questionnaire is composed of five questions that investigate the usability and experience with technology, rated on a scale of 1–5 (from 1 = very bad to 5 = very good). This questionnaire was administered to evaluate the participants’ usability and familiarity with technology, in particular with the 360° apparatus. Higher scores indicate greater usage of new technologies. It is an adaptation to Spanish from the *ad hoc* original scale (Schuemie, 2003), reviewed by a bilingual English-Spanish researcher.

#### Memory Task

The test is an adaptation of a previous work by Sauzeon et al. (2016) and measures the ability to remember the objects from the pictures viewed during the experiment. It is composed of two sub-tasks:

1. *Free recall tasks* where participants are asked to recall as many items as they can of the pictures they are shown on the immersive and non-immersive technologies. The free recall task was administered immediately after looking at the 360° VR environments. For each task, there are two scores: (1) *target score,* referring to the number of the target objects recalled for each picture and (2) *common score,* referring to the number of the recalled objects present in both pictures. Both scores are rated on a scale of 0–5 (0 = no item remembered, 5 = all items remembered).
2. *Recognition task* where participants have to recognize, from a written list, as many items as they can from the first pictures they viewed. The list consists of 10 varying objects presented in the two rooms and 5 common objects, for a total of 15 items. In this case, there are five interfering objects for both groups (five objects belong to other room) and 10 correct items. The recognition task was administered after a 10-min delay from the exposure. It is an adaptation to Spanish from the original scale (Negro-Cousa et al., 2019), reviewed by a bilingual English-Spanish researcher.

## Results

Statistical analyses were performed using the SPSS v.24.

### Sample Characteristics

The sample was composed of 17 females (40.5%) and 25 males (59.5%). The most represented age group was 18–24 years (*n* = 26; 61.9%), followed by 25–30 years (*n* = 10; 23.8%), and 34–55 years (*N* = 6; 14.3%). The level of education was undergraduate (*n* = 8; 19.1%), bachelor’s degree (*n* = 21; 50%), master’s degree (*n* = 7; 16.7%), and Doctor of Philosophy (*n* = 6; 14.3%). Analyses of Failure of Everyday Questionnaire (MFE-Q) indicated a total score of 12.82 (SD = 6.09); specifically for each factor: (1) memory activity *M* = 4.99 (SD = 2.67); (2) recognition *M* = 1.08 (SD = 1.44), and (3) memory communication *M* = 5.80 (SD = 3.31). Since the maximum score for the questionnaire is 56 (2 × 28 item), the average score of 12.82 indicated that the sample did not manifest particular memory deficit in daily life activities. The Simulator Sickness Questionnaire (SSQ) showed a moderate score of sickness for the immersive condition: nausea *M* = 9.82 (SD = 2.57) and oculomotor *M* = 10.02 (SD = 3.34). Moreover, participants had a good but not excellent experience with 360° technologies and VR in general *M* = 12.24 (SD = 1.90).

### Memory Task

After counterbalancing the groups, two paired *t*-tests were carried out to investigate whether the 360° immersive picture would facilitate free memory recollection in comparison to a non-immersive 360° condition. Results revealed no significant difference between conditions for both “target recall task” *t*(41) = −0.79, *p* = 0.429; 95% CI (−0.218, 0.504) and “common recall task” *t*(41) = −0.11, *p* = 0.906; 95% CI (−0.380, 0.428). The descriptive statistics are shown in Table 2.

**Table 2:** Descriptive statistics of recall task.

	*N*	Min.	Max.	*M* (SD)
Recall_Target (immersive)	42	1	5	3.95 (1.05)
Recall_Target (non-immersive)	42	1	5	3.80 (1.27)
Recall_Common (immersive)	42	1	5	3.95 (1.03)
Recall_Common (non-immersive)	42	1	5	3.92 (1.04)

Furthermore, independent *t*-test was carried out to evaluate the difference between conditions for the “recognition task.” Results showed a significant difference in “target recognition task” for the immersive condition *t*(40) = 2.09, *p* = 0.42; 95% CI (0.026, 1.403). However, there was no significant difference between groups on “common recognition task” *t*(40) = −0.18, *p* = 0.860; 95% CI (0.589, 0.493). The descriptive statistics are shown in Table 3.

**Table 3:** Descriptive statistics of recognition task.

	*N*	Min.	Max.	*M* (SD)
Recall_Target (immersive)	21	1	5	**4.10 (0.83)**
Recall_Target (non-immersive)	21	1	5	**3.38 (1.32)**
Recall_Common (immersive)	21	1	5	4.38 (0.80)
Recall_Common (non-immersive)	21	1	5	4.43 (0.92)

*Values in bold are statistically different between conditions*.

Two paired *t*-tests for each group were adopted to investigate the difference between immersive and non-immersive conditions to better evaluate the carryover effect. Group 1 (order immersive and non-immersive) showed a significant difference in “common recall task” but not for “target recall task” while Group 2 (order non-immersive and immersive) demonstrated a significant difference for the “target recall task” but not for “common recall task” (Table 4).

**Table 4:** Performance of participants in memory task for each condition.

		Immersive: *M* (SD)	Non-immersive: *M* (SD)	*t*	df	*p*
Group 1: immersive and non-immersive	Target	4.14 (1.017)	4.48 (1.062)	−1.503	20	0.149
	Common	4.19 (1.207)	3.62 (0.814)	2.168	20	0.042
	Total score	8.33 (1.238)	8.09 (1.220)	0.794	20	0.437
Group 2: non-immersive and immersive	Target	3.76 (1.117)	3.14 (1.315)	−2.540	20	0.020
	Common	3.71 (1.167)	4.24 (0.889)	2.057	20	0.053
	Total score	7.476 (2.040)	7.38 (1.774)	−0.336	20	0.741

Finally, moderation analyses showed that the specific subscale of MFE-Q did not moderate the effect of condition on memory tasks, both target and common recognition. Specifically, (1) recognition target: memory ability *F*(1, 42) = 0.123, *p* = 0.727, memory recognition *F*(1, 42) = 1.029, *p* = 0.317 and memory communication *F*(1, 42) = 0.088, *p* = 0.768; (2) recognition common: memory ability *F*(1, 42) = 0.478, *p* = 0.493, memory recognition *F*(1, 42) = 0.354, *p* = 0.556 and memory communication *F*(1, 42) = 0.087, *p* = 0.770.

## Discussion

The 360° camera is an innovative technology in the field of immersive VR. Recently, researchers have investigated the validity of 360° technology to assess episodic memory (Serino et al., 2017; Negro-Cousa et al., 2019). In this study, the general objective was to explore the feasibility of the 360° technology to assess memory ability. We compared 360° immersive and non-immersive setups in the memory task. Specifically, we hypothesized that participants in the 360° immersive condition could better perform than the 360° non-immersive condition in memory tasks.

This study confirmed the hypothesis that the immersive 360° panorama is more efficient in regard to memory ability than the non-immersive condition. Results showed no significant difference between immersive and non-immersive conditions for the “free recall task,” both in target and common tasks, which can be explained by the lack of any specific memory disorder among participants. All participants showed proficient memory ability in daily life activities, as evaluated by the *MFE-Q*. Further analyses also revealed that memory ability did not moderate the effect of condition on both target and common recognition tasks; however, the results in the “recognition task” were statistically significant for the target items. The recognition task administered after a 10-min delay from the exposure to the 360° picture can explain how the immersive exposure aids long-term memory encoding more than non-immersive exposure. The significance occurs only in the target task of the recognition test, namely for the recollection of the specific objects of the single room – A or B – but not for the common objects (Figure 1). The difference can be explained by the notions that participants may find it easier to recall objects presented twice than just a single exposure. Repeated exposures to the same stimuli increase the probability of recollection (Baddeley, 2012).

Finally, through the analysis of the independent Groups 1 and 2, results showed that Group 2, which encoded the 360° picture in the “non-immersive and immersive” order had significantly better recall scores in the second free recall task (after the presentation of 360° pictures through the immersive technology) rather than in the first free recall task (after the presentation of 360° pictures through non-immersive technology). These results support the idea that a more immersive condition was able to strengthen the memory ability for the specific objects, likely due to the sense of spatial presence elicited during the experience, as suggested by previous research (Serino and Repetto, 2018). The significance for the specific items is not present for the Group 1 order “immersive and non-immersive.” The realism provided by the 360° immersive environment and the relative sense of spatial presence – defined as “the sense of being there,” in a virtual or physical environment (Riva and Mantovani, 2012) – seem to play a role in strengthening the memory trace, which the non-immersive condition does not support. It can be hypothesized that the sense of presence vanishes when participants switch from an immersive to a non-immersive environment, while the sense of presence is strongest when participants switch from non-immersive to immersive environment. Makowski et al. (2017) and Schutte and Stilinović (2017) found that the sense of presence and the emotions experienced during the projection of a movie increased the capacity to recall specific frames watched during the experiment. In particular, the impact of emotions on memory recall was mediated by presence. In this study, the significance of remembered common objects occurred in Group 1 (“immersive and non-immersive”) but not in Group 2 (“non-immersive and immersive”). Even when objects are presented twice – in either condition – participants recalled them better only when they switched from immersive to non-immersive condition. One possible explanation is that the immersive condition – an innovative instrument for most participants – distracts the participants from what they had seen some seconds before through the headset (non-immersive condition). In the current study, familiarity with technology appears to play an important role. Most participants are accustomed to using non-immersive contents on their devices. An immersive experience could induce the sense of “awe” to participants, which could distract them from the research task (Chirico et al., 2017).

## Limitations and Conclusion

Two limitations were noteworthy from this study: (1) the sample size and (2) the independent control group. As the current study was only a preliminary research to test the feasibility of the immersive 360° technology for memory assessment, participants with no memory deficit were recruited, and the counterbalanced design emerged to be the most suitable strategy for indicating the differences between immersive and non-immersive conditions.

Future works should introduce measures for sense of presence to evaluate how it can moderate memory change. Larger sample sizes and a Randomized Control Trial design should also be implemented to better verify how the 360° immersive environment can be effectively used in memory assessment, as suggested by this study. Lastly, possible future works could be directed to populations with memory impairments to test if 360° technology could be an efficient rehabilitation tool to strengthen memory ability, as preliminary results of this study confirmed.

## Data Availability Statement

The datasets generated for this study are available on request to the corresponding author.

## Ethics Statement

The studies involving human participants were reviewed and approved by Ethics Committee of Valencia University (Spain). Registration number: H1543407702114. The patients/participants provided their written informed consent to participate in this study.

## Author Contributions

SV made substantial contribution to the conceptualization and design of the experiment, formal analyses, collection of the data and drafting the manuscript. EB made substantial contribution to the conceptualization and design of the experiment and in revising the manuscript. GR and RB made substantial contribution in revising the manuscript critically for important intellectual content. All authors provided final approval of the version to be published and agreed to be accountable for all aspects of the work as well as in ensuring that questions related to the accuracy or integrity of any part of the work are appropriately investigated and resolved.

### Conflict of Interest

The authors declare that the research was conducted in the absence of any commercial or financial relationships that could be construed as a potential conflict of interest.

## Abbreviations

VR: Virtual reality
HMD: Head-mounted display
RBMT: Rivermead behavioral memory test
MFE-Q: Failure of everyday questionnaire
SSQ: Simulator sickness questionnaire
